# Medication and Antibiotic Use Among Children Reported by an Online Convenience Sample of Parents in Serbia: A Cross-Sectional Survey

**DOI:** 10.3390/medsci14060624

**Published:** 2026-09-30

**Authors:** Ana Tomas Petrović, Nela Adžić, Nebojša Pavlović, Branislava Teofilović, Milica Paut Kusturica, Tihomir Dugandžija, Milica Vasilić, Nebojša Stilinović

**Affiliations:** 1 Department of Pharmacology, Toxicology and Clinical Pharmacology, Faculty of Medicine, University of Novi Sad, Hajduk Veljkova 3, 21000 Novi Sad, Serbia; ana.tomas@mf.uns.ac.rs (A.T.P.); milica.paut-kusturica@mf.uns.ac.rs (M.P.K.); nebojsa.stilinovic@mf.uns.ac.rs (N.S.); 2 Faculty of Medicine, University of Novi Sad, Hajduk Veljkova 3, 21000 Novi Sad, Serbia; 015855@mf.uns.ac.rs; 3 Department of Pharmacy, Faculty of Medicine, University of Novi Sad, Hajduk Veljkova 3, 21000 Novi Sad, Serbia; branislava.teofilovic@mf.uns.ac.rs; 4 Department of Epidemiology, Faculty of Medicine, University of Novi Sad, Hajduk Veljkova 3, 21000 Novi Sad, Serbia; tihomir.dugandzija@mf.uns.ac.rs; 5 Oncology Institute of Vojvodina, Put Doktora Goldmana 4, 21204 Sremska Kamenica, Serbia; 6 Pulmonary Institute of Vojvodina, Put Doktora Goldmana 4, 21204 Sremska Kamenica, Serbia; vasilicmica96@live.com

**Keywords:** self-medication, antibiotics, antimicrobial resistance, Serbia, drug utilization research

## Abstract

Background: Parents play a central role in medication use in children. Given the limited evidence in our setting, this study aimed to characterize and identify factors associated with antibiotic use and parent-directed medication without physician recommendation in Serbia. Methods: A cross-sectional online survey was conducted among an online convenience sample of parents or guardians of children younger than 12 years. The questionnaire collected sociodemographic data, information on medication and antibiotic use in the youngest child, sources of treatment recommendations, and parental knowledge and attitudes toward antibiotics. Factors associated with antibiotic use and parent-directed medication without physician recommendation were examined using multivariable modified Poisson regression with robust standard errors, with adjusted prevalence ratios (aPRs) and 95% confidence intervals reported. Results: A total of 1268 parents/guardians participated. Medication use during the previous six months was reported for 900/1268 children (71.0%, 95% CI: 68.4–73.4%), while 693/1268 (54.7%, 95% CI: 51.9–57.4%) had received antibiotics. During the last infection, the use of second- and third-generation cephalosporins (30.3%), co-amoxiclav (28.3%), and amoxicillin (20.8%) were most commonly reported. Higher prevalence of antibiotic use was associated with toddler age, kindergarten or school attendance, and chronic illness. Only five respondents (0.4%) reported parent-directed antibiotic administration without physician recommendation. In contrast, parent-directed medication without physician recommendation was reported for 514/900 children who had used medicines (57.1%, 95% CI: 53.9–60.3%), and higher prevalence was associated with preschool age, higher parental education, and parent being a healthcare professional, and lower prevalence with infancy and chronic illness. Conclusions: Medication use and parent-directed medication without physician recommendation were common, whereas parent-directed antibiotic use without physician recommendation was rarely reported among this online convenience sample of parents in Serbia.

## 1. Introduction

Parents play a critical, hands-on role in managing pediatric medication, acting as the primary decision-makers and administrators [1,2]. The use of over-the-counter (OTC) drugs as initial options by parents is common in the pediatric population, as many parents begin initial treatment of their children at home, either making decisions themselves or based on advice from social contacts, personal experience, or other information sources, without consulting a qualified physician [3]. In 2000, the World Health Organization (WHO) defined self-medication as the selection and use of medicines by individuals to treat self-recognized illnesses or symptoms [4]. Over time, the definition has broadened, with different studies describing diverse concepts included in the definition of self-medication. A recent review article suggested that self-medication can be regarded as a process in which OTC and prescription medications, herbal products, home-made remedies, nutritional supplements, and vitamins are purchased, selected, used, and consumed without professional advice, prescription, and supervision regarding indication, dose, and duration of treatment for chronic or acute symptoms [5]. Self-medication is a behavior in which the individual aims to treat self-recognized disorders or symptoms or tries to alleviate third parties’ ailments [5]. Responsible self-medication is an important component of self-care that may help manage minor ailments such as fever, cough, cold, and diarrhea and reduce unnecessary healthcare consultations and pressure on the health-care system [6]. However, inappropriate practices can expose individuals to medication-related harm, including therapeutic failure and adverse effects. This is particularly relevant in children, since drug pharmacokinetics and pharmacodynamics in pediatric age are different from those in adults [7].

Antimicrobial agents are one of the most commonly prescribed medications for children globally [8,9], and many of these prescriptions are unnecessary or inappropriate [10,11]. Overprescribing can occur amid diagnostic uncertainty, parental expectations for getting antibiotics, and potential pressure on prescribers [12,13]. Recent studies have revealed that parents also administer leftovers or previously prescribed antibiotics to their children, without consulting healthcare professionals, with notable differences in the prevalence of this practice in different countries [14,15]. Studies from different parts of the world indicate that between 22% and 70% of parents harbor misconceptions about rational antibiotic use and their effectiveness, leading them to use antibiotics without a prescription [16,17]. These practices have serious consequences, as using antibiotics when not indicated offers limited benefit while increasing the risk of antimicrobial resistance (AMR) and adverse effects. AMR is one of the greatest challenges of the 21st century and is recognized by the WHO as one of the top ten global public health threats [18]. A 2019 UN/WHO report attributed 700,000 deaths annually to AMR, a figure projected to increase to 10 million by 2050 under a worst-case scenario [19]. Beyond resistance, early-life antibiotic exposure has been linked to disruptions in gut microbiota and increased risks of atopic dermatitis, obesity, and inflammatory bowel disease [20]. Previous studies in Serbia have examined antibiotic-related knowledge and practices in the general population [21,22], identifying several misconceptions and patterns suggestive of antibiotic overuse. However, evidence on outpatient antibiotic use in children remains limited. A recent study on the effects of a multisectoral campaign for the Rational Use of Antibiotics implemented in Serbia found a 32.8% reduction in the number of prescribed systemic antibiotics per 1000 children between 2015 and 2021, representing one of the largest and most sustained reductions in pediatric antibiotic use in Europe [23]. However, prescription-based data cannot capture antibiotic self-medication or parental knowledge and attitudes toward antibiotic use. Moreover, little is known about the individual and family characteristics associated with medication use and parent-directed medication use without physician recommendation in Serbian children. Therefore, this study aimed to describe medication and antibiotic use among children and to examine factors associated with antibiotic use and parent-directed medication use without physician recommendation in Serbia.

## 2. Materials and Methods

### 2.1. Study Design and Setting

The cross-sectional study was conducted from 30 December 2023, to 19 January 2024, among parents of children younger than 12 years old in Serbia. The research instrument was an online survey questionnaire designed specifically for this study, created using an online platform (Google Forms (Google LLC, Mountain View, CA, USA)) and accessible from any device with an internet connection (PC or smartphone). Participants were recruited through convenience sampling by distributing the survey link in five Serbian Facebook groups intended for parents, with a combined reported membership exceeding 200,000. This figure does not correspond to unique individuals or actual survey reach because membership could overlap across groups. The survey link was shared three times in each group, and no paid advertising was used. All respondents confirmed that they lived in Serbia before proceeding to the questionnaire. We did not collect specific residential locations within Serbia. Participation was voluntary and anonymous. Eligible respondents were parents or legal guardians with at least one child younger than 12 years in the same household. Where respondents had more than one child, all medication-use questions referred to the youngest child in the household. Based on the previously reported annual prevalence of antibiotic prescribing of 54% in children in Serbia [24], with a 95% confidence level and a 5% margin of error, the minimum required sample size calculated was 382 participants. Because this prevalence estimate was based on data collected about 10 years earlier and referred to yearly prevalence of antibiotic use, and the present study assessed antibiotic use during the previous six months, recruitment was intentionally continued to account for uncertainty in the expected prevalence and to improve the precision of the estimates. A total of 1328 survey responses were received. Of these, 58 were excluded because respondents did not answer the question on medication use in their youngest child. Responses were manually inspected for identical or near-identical patterns across demographic and questionnaire items. After manual review, we identified two submissions as duplicates and excluded one from each pair, retaining one record from each duplicate pair. The final sample contained 1268 respondents, corresponding to 95.5% of the submitted responses. The numbers of individuals who viewed the survey, provided consent, or started but did not submit a response were not separately recorded and view, participation, and completion rates could not be calculated.

### 2.2. Survey Tool

The questionnaire was specifically developed for this study. The questionnaire was developed in Serbian and underwent expert review before data collection. Four experts from the research team with complementary expertise in clinical pharmacology, epidemiology, public health, and pharmacy practice independently reviewed the draft questionnaire. The expert review focused on the relevance of the questionnaire domains and individual items to the study objectives, scientific accuracy, clarity and comprehensibility of the wording, appropriateness of the response options, and suitability for the target population. The assessment was qualitative and based on expert judgment. No formal content-validity index was calculated. Revisions were made to improve wording, clarify the reference period for selected questions, and refine response options. The revised questionnaire was then pilot-tested on a sample of 30 participants. Based on this pilot testing, minor wording adjustments were made to improve comprehensibility, without changing the intended meaning of the items. No formal cognitive interviews or psychometric reliability assessments were conducted. Data from the pilot-test participants were not included in the final analysis. In Section 1 of the survey, respondents were informed about the study objectives, as well as privacy, data protection, and voluntary participation. Section 2 collected information on the respondent’s relationship to the child, ensuring that only parents or guardians participated. Section 3 gathered demographic data of parents/guardians, including sex, age, marital status, highest level of education, household size, number of children in the household, and whether they or their family member was a healthcare professional. Section 4 gathered data on the occupations of respondents who identified as healthcare workers.

The main part of the questionnaire focused on the youngest child in the family, including sex, age, attendance at kindergarten or school, presence of chronic illnesses requiring daily medication, and whether the child had taken any medication in the past six months.

Based on the responses, participants were categorized into two groups: Those who answered “NO” to the last question proceeded to a section on attitudes and practices of antibiotic use and their interest in education on rational medication use. Those who answered “YES” were asked to provide details on the type of medication and source of recommendation for the following categories: fever-reducing medication, nose drops, cough syrups, immune system supplements, inhalations, vitamins and minerals, and vaccines. They were then asked whether their child has taken antibiotics in the past 6 months. Participants who reported that their youngest child had taken antibiotics in the past six months were directed to a section that gathered information on the antibiotics used during the last infection: antibiotic used, times antibiotics were used in the previous 6 months (1, 2, or 3 or more times), indications (multiple indications could be reported), and who recommended antibiotics to their child. To allow for easier recall of antibiotics used, parents were offered licensed and international non-proprietary names (in brackets) of all antibiotics registered for use in children in Serbia (grouped into subcategories), as well as an open-text response option, if the drug their child used was not listed. In the next part of the survey, respondents indicated whether each of the 10 statements about parental attitudes, knowledge and practices regarding antibiotics was true or false.

### 2.3. Ethical Approval

The study was approved by the Ethics Committee for Clinical Research on Humans, Faculty of Medicine, University of Novi Sad, under reference number: 01-39/123/1.

### 2.4. Variables

The age of parents was determined based on the year of birth they provided, and the age of children was reported in years or months for children under 1. Based on the question on the age of the youngest child, child age was categorized as Infant (<1 year), Toddler (1 to <3 years), Preschool child (3 to <6 years), and Child (6 to <12 years). During the analyses, parental education was grouped into three categories: primary or secondary education, university education, and postgraduate education to address sparse observations in the primary education category. Antibiotics were classified based on the international non-proprietary names into penicillins, beta-lactam/beta-lactamase-inhibitor combination, 1st generation cephalosporins, 2nd and 3rd generation cephalosporins, macrolides, and phosphonic acid derivatives. Specific drugs within categories were penicillins (amoxicillin), 1st generation cephalosporins (cephalexin, cefadroxil), 2nd and 3rd generation cephalosporins (cefixime, cefprozil, cefuroxime, cefpodoxime), macrolides (azithromycin, clarithromycin), phosphonic acid derivatives (oral fosfomycin). The World Health Organization’s AWaRe classification of antibiotics, which groups antibiotics into Access, Watch, and Reserve categories, based on clinical importance and the risk of promoting resistance, was also used to describe patterns of use [25].

### 2.5. Outcome

The primary outcome was medication use, and secondary outcomes were antibiotic use and parent-directed medication without physician recommendation. Medication use was coded as “yes” or “no” based on the answer to the question “has your child used any medication in the previous 6 months?”. The same was done for the question on antibiotic use. To determine the prevalence of parent-directed medication without physician recommendation in children who reported medication use (n = 900), a new dichotomous variable was created, parent-directed medication without physician recommendation (yes–no), based on questions from the part of the questionnaire on how drugs were obtained. The questionnaire recorded recommendation sources for each of the following product categories: fever-reducing medicines, nasal drops, cough syrups, immune system supplements, inhaled products, vitamins and minerals, and vaccines. Parent-directed medication without physician recommendation was coded as “yes” if at least one product was administered based on personal choice, information from the internet, or recommendations from friends or family. No pharmacist recommendations were reported. Respondents reporting both physician-recommended and parent-selected products, including multiple recommendation sources for the same product category, were classified as “yes.” All five respondents who reported antibiotic use without physician recommendation also reported parent-directed medication without physician recommendation for other surveyed products and were therefore already classified as “yes”. In Serbia, prescribing rights for human medicines are limited to doctors of medicine and dental medicine, and the parent-directed medication without physician recommendation variable was coded as “no” when all reported products were administered exclusively on a doctor’s recommendation. Information was sufficient to classify all 900 respondents. This operational definition captured the source of the treatment recommendation, and the surveyed categories included medicines and other health products and did not permit differentiation between prescription and non-prescription medicines or between medicated and saline nasal or inhaled products. Supplements, vitamins and minerals, and vaccines were included within the broad self-medication outcome definition and should be considered when interpreting the estimates [5]. The self-medication variable captured the source of the treatment decision rather than its appropriateness, and parent-directed medication without physician recommendation as defined in this study should not automatically be interpreted as unsafe or inappropriate use.

### 2.6. Statistical Analysis

Statistical analyses were performed using IBM SPSS Statistics software (version 22.0 for Windows, IBM Corp., Armonk, NY, USA). Categorical variables were reported as frequencies and percentages, and continuous variables as means and standard deviations or medians and interquartile ranges. Prevalence estimates were reported with 95% confidence intervals (CIs) calculated using the Wilson score method. Two outcomes were examined: antibiotic use during the preceding six months and parent-directed medication without physician recommendation. Because only five respondents reported antibiotic use without a physician recommendation, no separate regression analysis was performed. The antibiotic-use analysis included all 1268 participants, of whom 693 had the outcome, and the parent-directed medication without physician recommendation analysis was restricted to the 900 children who received at least one medication during the preceding six months, out of which 514 had the outcome. Associations between parental and children characteristics and each outcome were initially explored using univariable regression analyses (Supplementary Tables S2 and S3). Given the high prevalence of both outcomes, multivariable modified Poisson regression with a log link and robust sandwich standard errors was used to estimate adjusted prevalence ratios (aPRs) and 95% CIs. Covariates were selected based on univariate analyses, but also to account for child characteristics, parental characteristics, and family context potentially associated with medication use. The principal antibiotic-use model included child age, child sex, parental education, parental healthcare-professional status, kindergarten or school attendance, chronic illness requiring regular medication in the child, number of children in the family, and parental age. The principal parent-directed medication without physician recommendation model included child age, child sex, parental education, parental healthcare-professional status, kindergarten or school attendance, chronic illness requiring regular medication in the child, household size, and parental age. Marital status was excluded from the multivariable models because of sparse categories and unstable estimates. Multicollinearity was assessed using variance inflation factors (VIFs) obtained from auxiliary linear regression models incorporating the predictors of the principal models. In the antibiotic-use analysis (n = 1268), VIFs ranged from 1.005 to 2.017, with the highest value observed for kindergarten or school attendance. When the number of children and household size were included simultaneously, VIFs for these family-composition variables increased to 4.952–6.390. Consequently, these variables were not included simultaneously in the principal models and were examined separately in sensitivity analyses. In the principal parent-directed medication without physician recommendation analysis (n = 900), VIFs ranged from 1.01 to 1.78, with the highest value observed for kindergarten or school attendance. When household size and number of children were included simultaneously, VIFs for their indicator variables increased to 4.60–5.87, and these were evaluated in separate model specifications, with household size retained in the principal parent-directed medication without physician recommendation model. Binary logistic regression using the same covariates as the respective principal modified Poisson models was additionally performed, with adjusted odds ratios (aORs) and 95% CIs reported. Results of the sensitivity analyses and model diagnostics performed for both outcomes are available in Supplementary Tables S4–S11. Model diagnostics included examination of fitted prevalences, standardized Pearson residuals, leverage values, and Cook’s distance. Calibration of the logistic regression models was assessed using the Hosmer–Lemeshow goodness-of-fit test. All statistical tests were two-sided, with *p* < 0.05 considered statistically significant.

## 3. Results

### 3.1. Characteristics of Respondents

A total of 1268 parents or guardians participated in this study conducted on an online convenience sample in Serbia. The mean age of respondents was 35.6 ± 5.2 years. Most respondents were women (n = 1227; 96.8%), married (n = 1167; 92.0%), and highly educated (Table 1). Most households had four members (n = 615; 48.5%). Overall, 165 respondents (13.0%) were healthcare professionals, and among the 155 respondents who specified their healthcare profession, the majority listed being technicians/nurses and nursing-related professionals (n = 64; 41.3%), followed by physicians (n = 35; 22.6%), pharmacists (n = 23; 14.8%), and dentists or dental technicians (n = 14; 9.0%). A total of 95 respondents reported having a healthcare professional in their family. The most frequently reported family-member profession was medical technician/nurse (n = 43; 45.3%), followed by physician (n = 32; 33.7%), dentist (n = 9; 9.5%), and pharmacist (n = 4; 4.2%).

Table 2 shows characteristics of children for whom the parents completed the survey on medication use. The median age of the youngest child was 3 years (interquartile range: 2–4 years). The sex distribution was almost equal. Most children were preschool children (n = 553; 43.6%) and toddlers (n = 430; 33.9%). A total of 1023 children (80.7%) attended kindergarten or school, while 41 children (3.2%) had a chronic disease requiring chronic medication use.

### 3.2. Medication Use in Children

In the previous six months, 900/1268 children (71.0%, 95% CI: 68.4–73.4%) had used at least one medication (Table 3). Antipyretics were the most frequently used medicines. When medication use was analyzed by source of recommendation, all vaccines and almost all antibiotics were administered following a physician’s recommendation, while parent-directed medication without physician recommendation was mainly related to symptomatic treatment. Recommendation sources by product category are presented in Supplementary Table S1. Most respondents expressed a high level of interest in receiving professional education on the safe use of medicines in children. Most parents/guardians gave the highest score (5) to the statement “I would like to have the opportunity to receive information from healthcare professionals about the safe use of medicines in children” (63.5%). An additional 11.7% of respondents selected score 4 (Supplementary Figure S1).

### 3.3. Antibiotic Use in Children

Results of the univariate analyses are available in Supplementary Table S2. Antibiotic use during the preceding six months was reported for 693/1268 children (54.7%, 95% CI: 51.9–57.4%), with complete data on the outcome for all 1268 participants. In the modified Poisson regression, child age was significantly associated with antibiotic use. Compared with toddlers, antibiotic-use prevalence was lower among infants (aPR = 0.48, 95% CI: 0.34–0.68; *p* < 0.001), preschool children (aPR = 0.85, 95% CI: 0.76–0.95; *p* = 0.004), and school-aged children (aPR = 0.63, 95% CI: 0.52–0.77; *p* < 0.001). Higher antibiotic-use prevalence was associated with kindergarten or school attendance (aPR = 1.20, 95% CI: 1.01–1.43; *p* = 0.041) and chronic illness requiring regular medication (aPR = 1.51, 95% CI: 1.31–1.75; *p* < 0.001). The number of children in the family was associated with antibiotic use overall (*p* = 0.036). Compared with one-child families, antibiotic-use prevalence was lower in families with two children (aPR = 0.88, 95% CI: 0.78–0.98; *p* = 0.018), while the association for families with three or more children did not reach statistical significance (aPR = 0.86, 95% CI: 0.75–1.00; *p* = 0.051). Child sex, parental education, parental healthcare-professional status, and parental age were not significantly associated with antibiotic use after adjustment for the other variables (Table 4). Model diagnostics identified five observations (0.4%) with fitted prevalence exceeding 1 in the principal modified Poisson model, and two observations (0.2%) exceeded the Cook’s distance screening threshold of 4/N. All standardized Pearson residuals were within ±3. Detailed diagnostic results are presented in Supplementary Table S6. Sensitivity analyses showed consistent findings across alternative model specifications (Supplementary Tables S4–S7). The associations of child age and chronic illness with antibiotic use were maintained across the modified Poisson models that included these variables. The reduced model produced estimates similar to those of the principal model, and excluding kindergarten or school attendance resulted in only modest changes in the child-age estimates. However, the estimated association with kindergarten or school attendance was sensitive to adjustment for child age. When child age was excluded, the aPR for attendance increased from 1.20 (95% CI: 1.01–1.43) to 1.41 (95% CI: 1.20–1.67). Replacing the number of children in the family with household size did not alter the estimates for the remaining predictors. In this alternative model, children living in households with four members (aPR = 0.86, 95% CI: 0.77–0.96; *p* = 0.008) or five or more members (aPR = 0.85, 95% CI: 0.74–0.97; *p* = 0.015) had a lower prevalence of antibiotic use than children living in households with three or fewer members (Supplementary Tables S4–S6). Multivariable logistic regression showed consistent directions of association (Supplementary Table S7).

**Figure 1:**
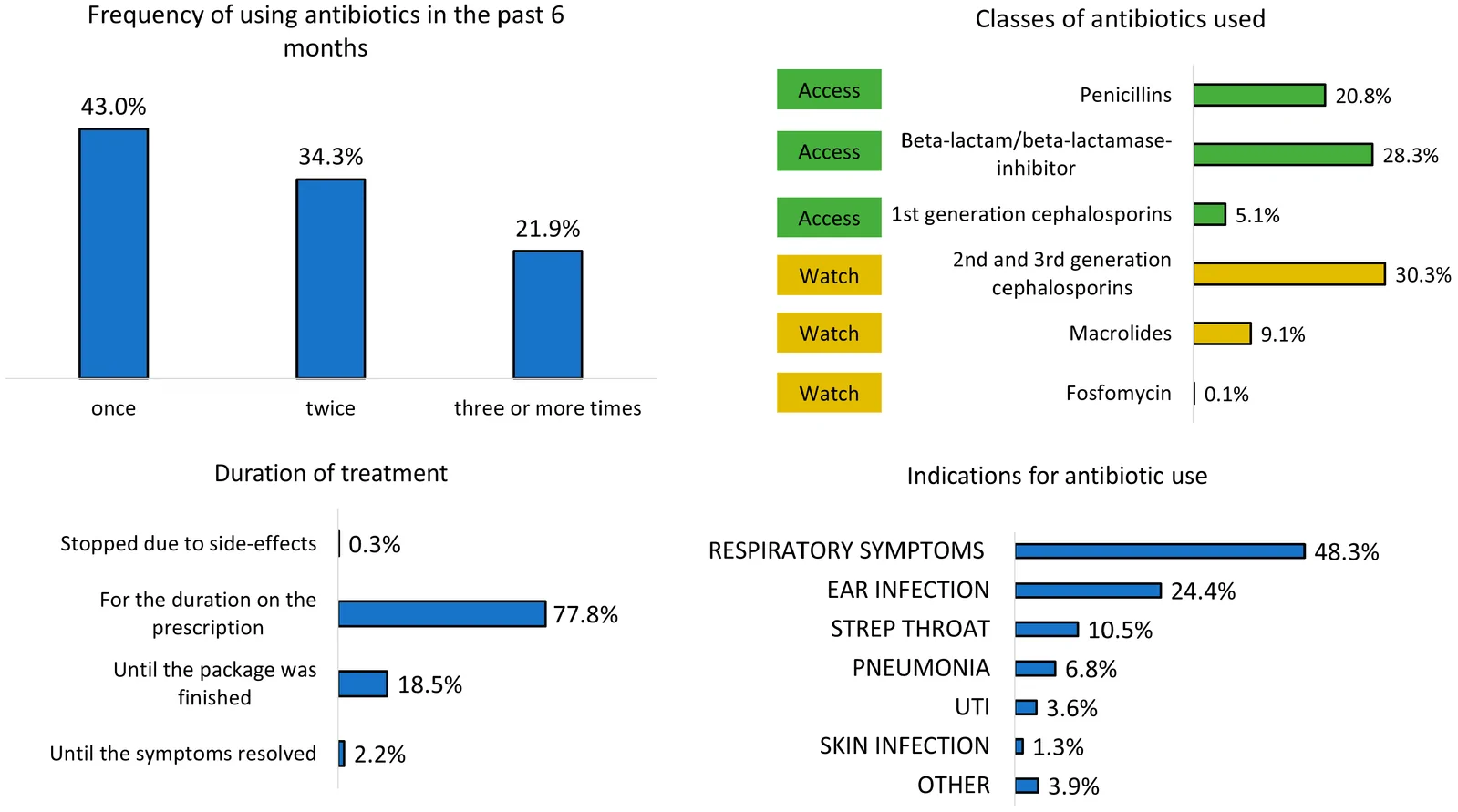
Frequency of antibiotic use during the previous 6 months and classes, indications, and duration of treatment during the last infection for respondents who reported their child had received antibiotics in the previous 6 months. Percentages were calculated using all 693 respondents reporting antibiotic use as the denominator. Multiple indications could be reported. Access and Watch indicate WHO AWaRe categories. Antibiotic categories included penicillins (amoxicillin), beta-lactam/beta-lactamase inhibitor combinations (co-amoxiclav), first-generation cephalosporins (cephalexin, cefadroxil), second- and third-generation cephalosporins (cefixime, cefprozil, cefuroxime, cefpodoxime), macrolides (azithromycin, clarithromycin), and fosfomycin. Not displayed: missing responses for frequency (n = 5; 0.7%), treatment duration (n = 9; 1.3%), and respondents unable to identify the antibiotic used (n = 44; 6.4%).

Among 693 respondents who reported antibiotic use in children in the previous 6 months, 298/693 (43.0%) of children had used antibiotics once, 238/693 (34.3%) had used them twice, and 152/693 (21.9%) had used antibiotics three or more times during the observed period (Figure 1). Frequency data were missing for five respondents. The most frequently reported indications for antibiotic use were respiratory symptoms (runny nose, cough, throat infection) (n = 335/693; 48.3%) and ear infection (n = 169/693; 24.4%). Respondents could report multiple indications. The most commonly used antibiotics for the most recent infection were second- and third-generation cephalosporins (n = 210/693; 30.3%), followed by co-amoxiclav (n = 196/693; 28.3%) and amoxicillin (n = 144/693; 20.8%). Macrolides (63/693; 9.1%), first-generation cephalosporins (35/693; 5.1%), and fosfomycin (1/693; 0.1%) were less common. Some respondents (44/693; 6.4%) could not specify the antibiotic used. Access group antibiotics accounted for 54.1% (375/693) of antibiotics reported for the most recent infection. Most parents reported that antibiotics were administered for the number of days prescribed by the physician (n = 539/693; 77.8%), 128/693 parents (18.5%) reported giving antibiotics until the package was finished, while 15/693 parents (2.2%) stopped treatment when the child’s symptoms resolved. Antibiotics were discontinued because of poor tolerability in 2/693 cases (0.3%). Treatment-duration data were missing for 9 respondents.

Parent-directed antibiotic use without physician recommendation was recorded in only 5 respondents (5/693, 0.7%), corresponding to 5/1268 participants (0.39%). During the observed period, parents or guardians self-administered antibiotics to their child once in 3/5 (60.0%) or twice in 2/5 (40.0%). Reasons included cough in three cases (3/5, 60.0%), fever in one case (1/5, 20.0%), and a combination of symptoms in one case (1/5, 20.0%). Reasons for not consulting a doctor included having enough experience to judge if their child needed antibiotics 4/5 (80.0%) and traveling 1/5 (20.0%). Sources of antibiotics included unopened packages kept in the household “just in case” in four cases (4/5, 80.0%) and leftover antibiotics from a previous unfinished treatment course in one case (1/5, 20.0%). All self-administered antibiotics were selected based on the child previously being prescribed the same antibiotic for a disease presenting with similar symptoms (5/5, 100.0%).

**Figure 2:**
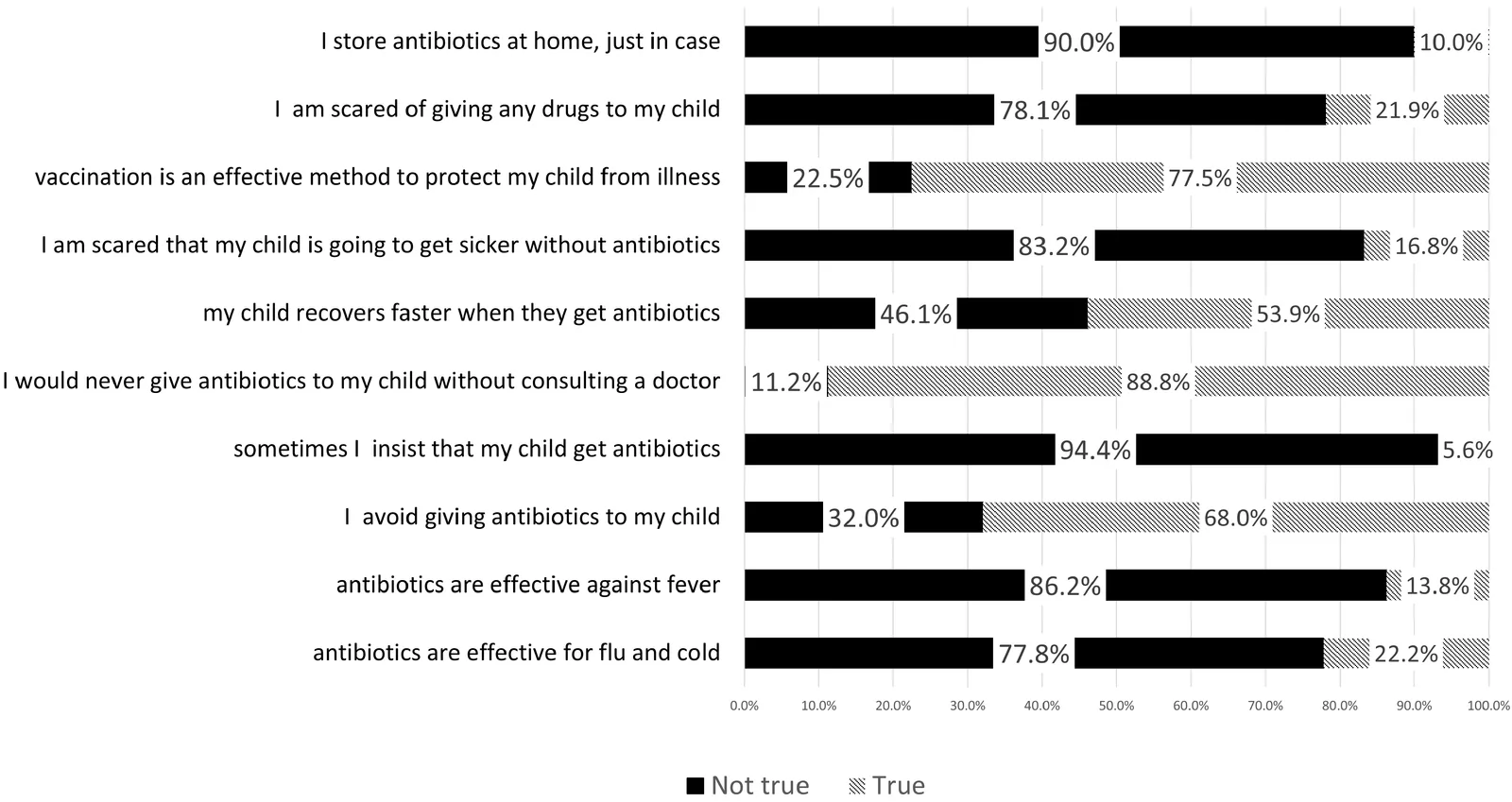
Parental attitudes and knowledge regarding antibiotic use in children (n = 1268).

Parental attitudes and knowledge regarding antibiotic use in children are presented in Figure 2. Most reported that they would never give antibiotics to their child without consulting a doctor (88.8%) and that they do not insist that their child receives antibiotics (94.4%). Similarly, most respondents stated that they do not store antibiotics at home “just in case” (90.0%). Most parents also demonstrated correct knowledge regarding the indications for antibiotics. A total of 77.8% did not consider antibiotics effective for flu and colds, while 86.2% did not consider antibiotics effective against fever. More than half (53.9%) believed that their child recovers faster when treated with antibiotics, but most respondents expressed caution in antibiotic use, with 68.0% reporting that they avoid giving antibiotics to their child.

### 3.4. Parent-Directed Medication Without Physician Recommendation (Self-Medication)

Results of the univariate analyses are available in Supplementary Table S3. Parent-directed medication without physician recommendation was reported for 514/900 children (57.1%,95% CI: 53.9–60.3%), corresponding to 514/1268 participants overall (40.5%). The outcome was analyzed for 900 children who had received at least one medication during the past six months, and in the multivariable modified Poisson regression model, child age, parental education, parental healthcare-professional status, and chronic illness were significantly associated with parent-directed medication without physician recommendation (Table 5). Compared with toddlers, infants had a lower prevalence of parent-directed medication without physician recommendation (aPR = 0.65, 95% CI: 0.44–0.97; *p* = 0.036), whereas preschool-aged children had a higher prevalence (aPR = 1.18, 95% CI: 1.04–1.35; *p* = 0.014). No statistically significant difference was observed between school-aged children and toddlers (aPR = 1.04, 95% CI: 0.84–1.28; *p* = 0.731). Compared with parents with primary or secondary education, those with university (aPR = 1.23, 95% CI: 1.06–1.42; *p* = 0.007), and postgraduate education (aPR = 1.27, 95% CI: 1.07–1.52; *p* = 0.008) had a higher prevalence of parent-directed medication without physician recommendation. Healthcare professional status was associated with higher (aPR = 1.28, 95% CI: 1.12–1.46; *p* < 0.001), and chronic illness with a lower (aPR = 0.60, 95% CI: 0.40–0.91; *p* = 0.016) prevalence of parent-directed medication without physician recommendation. Child sex, kindergarten or school attendance, household size, and parental age were not significantly associated with self-medication. Sensitivity analyses supported the findings (Supplementary Tables S8–S11). The associations of parental education, parental healthcare-professional status, and chronic illness with parent-directed medication without physician recommendation were maintained across the modified models. The estimated association with kindergarten or school attendance was sensitive to adjustment for child age. When child age was excluded, attendance was associated with a higher prevalence of parent-directed medication without physician recommendation (aPR = 1.33, 95% CI: 1.10–1.60; *p* = 0.003), whereas the corresponding association was not statistically significant in the principal model (aPR = 1.07, 95% CI: 0.87–1.32; *p* = 0.540). Detailed results are presented in Supplementary Tables S8–S10. Multivariable logistic regression yielded consistent directions of association (Supplementary Table S11). Model diagnostics identified no observations exceeding the Cook’s distance screening threshold of 4/900 in the principal parent-directed medication without physician recommendation model; no fitted prevalences exceeded 1, and all standardized Pearson residuals were within ±3.

## 4. Discussion

Antibiotics are among the most commonly prescribed medicines in children. In recent years, antibiotic prescribing rates in children have decreased in Western European countries [26]. Country-specific campaigns promoting prudent antibiotic use have probably contributed substantially to this change in practice. Historically, estimates suggest that up to 50% of antimicrobial prescriptions for children may be unnecessary [27]. In the present study, 693 respondents (54.7%, 95% CI: 51.9–57.4%) reported that their youngest child had taken antibiotics during the previous six months. Among children who had taken antibiotics (693), 43.0% had taken them once, 238 (34.3%) twice, and 152 (21.9%) three or more times during the past six months. This estimate is numerically similar to a Serbian study of systemic antibiotic prescriptions in pediatric primary care, which found an annual prevalence of approximately 54% [24]. However, direct comparison is limited by differences in observation periods and data sources. In the present study, the reported rate of parent-directed antibiotic self-medication was low, with less than 1% of parents/guardians reporting giving antibiotics to their children without physician recommendation, which contrasts with results of a systematic review where the prevalence of self-medication with antibiotics in children ranged from 8% in Europe to 34% reported in the Middle East [14]. This low prevalence observed in our study should be interpreted cautiously given the online convenience sampling and specific respondent characteristics. Most respondents were women (96.8%), 68.5% had university or postgraduate education, and 13.0% were healthcare professionals. Despite anonymous participation, social desirability bias may have contributed to underreporting of antibiotic use without physician recommendation. The extent of such underreporting cannot be determined. The small number of reported cases precluded a separate regression analysis of factors associated with antibiotic self-medication. Accordingly, this finding should not be interpreted as evidence that parent-directed antibiotic use without physician recommendation is similarly uncommon among all parents in Serbia.

The most commonly used antibiotics during the most recent infection were second- and third-generation cephalosporins and co-amoxiclav. In a study by Youngster and colleagues, analyzing antibiotic use in children across six countries, the most commonly used antibiotics in Norway were natural penicillins (64.8%) [9]. A recent study on the effects of a multisectoral campaign for the Rational Use of Antibiotics implemented in Serbia documented an increase in the relative contribution of third-generation cephalosporins and macrolides in antibiotic utilization in children despite a 32.8% reduction in the number of prescribed systemic antibiotics per 1000 children between 2015 and 2021 [23]. In line with these findings, in the present study, second- and third-generation cephalosporins, classified as Watch antibiotics, and co-amoxiclav, classified as an Access group antibiotic, accounted for the largest share of reported antibiotic use for the most recent episode. The issue of use of broad-spectrum antibiotics has been a long-standing problem in Serbia [28,29]. Watch group antibiotics should be used with caution because they have a high potential to contribute to antimicrobial resistance. Therefore, improving the quality of use of this antibiotic group should remain a focus of antimicrobial stewardship programs in Serbia [23,28]. In the present study, the most frequently reported indications for antibiotic use were respiratory symptoms, such as runny nose, cough, and throat pain (48.3%), and ear infection (24.4%). Previous studies indicate that a substantial share of pediatric antibiotic prescriptions are issued for acute respiratory tract infections, even though most of these infections are viral and self-limiting and antibiotics provide no benefit [30,31,32]. However, indications in the present study were parent-reported and not confirmed in medical records, so the interpretation of these findings should be done with caution. Parent-reported symptoms cannot be used to distinguish viral from bacterial infections or identify clinical findings that may have justified treatment, and the reported indications cannot be used to imply appropriateness or inappropriateness of antibiotic use.

Child and parent characteristics, and family context were associated with reported pediatric antibiotic use identified through an online convenience sample of parents in Serbia. In the multivariable modified Poisson regression, antibiotic-use prevalence was higher among toddlers than among infants, preschool children, and school-aged children. Higher prevalence was also associated with chronic illness in the child and kindergarten or school attendance. Compared with children from single-child families, those from families with two children had a lower prevalence of antibiotic use. The higher prevalence observed among toddlers is consistent with previous pediatric drug-utilization studies. Earlier national data from Serbia showed the highest prevalence of antibiotic prescribing among children aged 2–23 months [24], while studies from other European settings have similarly reported an association between children’s age and antibiotic use [33,34,35]. The higher prevalence among children attending kindergarten or school may reflect increased exposure to respiratory and other communicable diseases, consistent with previous studies [36,37]. Likewise, the substantially higher prevalence among children with chronic illness may be related to greater susceptibility to infections or more frequent healthcare contacts [38]. The lower prevalence of antibiotic use among children from households with two or more children should be interpreted cautiously, as previous studies have reported inconsistent associations between sibling status and pediatric antibiotic use [39]. For families with three or more children, the estimated prevalence was also lower, but the association was not statistically significant. Because medication-use questions referred specifically to the youngest child, differences in the age distribution of the youngest child across families of different sizes may partly explain this association. These children may differ from their older siblings in infection frequency, medication exposure, and kindergarten or school attendance. Adjustment for child age attenuated, but did not eliminate, the association, suggesting that other family-level factors may also contribute. The age distribution of the youngest child is related to family composition, which may partly explain the observed association between number of children and antibiotic use. Although the principal model adjusted for child age and attendance, and a sensitivity analysis examined household size as an alternative measure of family composition, residual confounding and the implications of selecting only the youngest child cannot be excluded. The observed association should therefore not be interpreted as an independent protective effect of larger family size.

Most parents reported administering antibiotics for the number of days prescribed by the physician, but 18.5% reported giving antibiotics until the package was finished, while 2.2% stopped treatment when symptoms resolved. This either suggests that some parents may misunderstand the difference between prescribed treatment duration and package size or that finishing the package may have coincided with completing the prescribed course [40]. Because prescribed doses and treatment duration were not verified, these practices could not be reliably classified as adherent or non-adherent to the recommended treatment course. Parental attitudes and knowledge about antibiotic use were generally appropriate. Most respondents did not believe antibiotics are effective against flu, colds, or fever, and most stated that they would not give antibiotics without consulting a doctor. These findings are consistent with the low level of reported antibiotic self-medication. A study from Italy that included 6625 parents from all Italian regions reported that 76% of parents knew that antibiotics are effective only against bacteria, while 92.9% knew that antibiotics have no direct effect on fever. Furthermore, 93% of parents knew that excessive antibiotic use can cause bacterial resistance, and 84.7% knew that they could actively contribute to combating antimicrobial resistance. Approximately two-thirds of participants (66.1%) received information about antimicrobial resistance from their family pediatrician [12]. However, the study identified some potential misconceptions. More than half of parents believed their child recovers faster when treated with antibiotics, and a smaller proportion feared the child would become sicker without them. These beliefs may shape expectations for antibiotic prescribing during medical consultation. Because the statement did not specify the viral or bacterial etiology of infection, this response should not be interpreted as unequivocally incorrect; rather, it may indicate a generalized perception of antibiotic benefit across childhood illnesses. Previous studies on antibiotic knowledge in Serbia found discrepancies between knowledge and practices, where higher knowledge does not equate to more rational antibiotic use [22,41]. To communicate the importance of not using antibiotics when they are not necessary, education should address potential acute adverse effects of antibiotics, i.e., gastrointestinal upset, allergies, skin rash, but also potential longer-term consequences: observational studies have associated early-life antibiotic exposure with subsequent asthma, allergic diseases, and obesity, although these associations do not establish causality and may be influenced by underlying infections and other confounding factors [42,43,44,45,46].

Parent-directed medication without physician recommendation was common in the present study, reported for 514 of the 900 children who had used medication during the preceding six months (57.1%; 95% CI: 53.9–60.3%). In the multivariable modified Poisson model, parent-directed medication without physician recommendation prevalence was lower among infants and higher among preschool-aged children compared with toddlers, but the findings did not indicate a consistent increase in self-medication with increasing child age. Parents with university or postgraduate education had a higher prevalence of parent-directed medication without physician recommendation than those with primary or secondary education. Being a healthcare professional was associated with approximately 28% higher prevalence, and chronic illness requiring regular medication was associated with approximately 40% lower prevalence after adjustment. No studies from Serbia are available for direct comparison. Still, previous studies in other countries have reported more frequent parental administration of over-the-counter medicines among older children [47], and associations between parental education and self-medication in children. However, their direction has varied across settings [14,48]. Similar to our findings, a study from Montenegro reports on maternal healthcare employment as a factor associated with greater odds of self-medication in children [49], while a Spanish study found that children without chronic illness were more likely to receive medicines without prescription [47]. High reported interest in education on medication use in children suggests an opportunity for educational interventions in our setting. It is important to note that the associations identified in the present study do not establish whether parent-directed use was inappropriate or appropriate; rather, they identify a potential target audience and characteristics associated with this practice. Evidence from the literature points to several problems parents encounter when administering medication to children, where education could be beneficial—i.e., lack of knowledge regarding the dose, dose frequency, and duration of treatment, and problems with adequate dose measurement. Medications for children are commonly dosed by weight or body surface area, and parents need to distinguish between measurement units and liquid medication volumes to administer an appropriate dose [50]. We believe educational programs for parents in our setting should focus on safety, product selection, appropriate dosing, and when to seek professional advice, while antibiotic-focused education could address identified potential misconceptions and inform parents about acute and long-term potential adverse effects of antibiotic use in childhood. A recently published systematic review of educational interventions for parents of pediatric patients found that structured educational interventions combining counseling, health literacy-informed materials, and standard dosing tools significantly improved caregiver medication knowledge and reduced dosing errors [51].

This study has several limitations that need to be mentioned. First, data were self-reported and may be affected by recall bias or social desirability bias, particularly for antibiotic self-medication. Reported symptoms, medicines, doses, and treatment duration were not verified against prescriptions or medical records; therefore, we could not reliably assess treatment appropriateness and adherence. Second, the survey was distributed online, which favored participation by more educated and digitally active parents, resulting in a sample that was highly skewed toward highly educated women, with high representation of health-care professionals. This online convenience sample should not be considered representative of all parents or children in Serbia. Counts of survey initiation without submission could not be recorded, preventing calculation of participation and completion rates and assessment of non-response bias. Although manual review removed two apparent duplicate submissions, this does not definitively exclude duplicate responses. Third, medication-use questions referred only to the youngest child in each household. The relationship between the youngest child’s age and family composition may partly explain the association between number of children and antibiotic use. Adjustment for child age and attendance, together with sensitivity analyses using household size, does not fully resolve this limitation; the findings should not be interpreted as an independent protective effect of larger families. Fourth, although the questionnaire underwent expert review and pilot testing for clarity and comprehensibility, formal cognitive interviewing and psychometric reliability assessment were not performed. Finally, the cross-sectional design does not allow for causal interpretation of factors associated with antibiotic use and self-medication.

## 5. Conclusions

In conclusion, medication use among children reported by an online convenience sample of parents in Serbia was common, and antibiotic use was reported for more than half of children in the previous 6 months. The use of broad-spectrum antibiotics remains a concern. Parent-directed medication without physician recommendation was frequent for drugs used for symptom relief and vitamins and minerals. Given the high parental interest in guidance on safe and rational medication use in children, educational programs delivered by healthcare professionals should be considered.

## Figures and Tables

**Table 1:** Demographic and household characteristics of participating parents/guardians in an online convenience sample of parents in Serbia (n = 1268).

Characteristic	N	%
Sex		
Male	41	3.2
Female	1227	96.8
Education		
Primary school	8	0.6
High school	391	30.8
University degree	643	50.7
Master’s or doctoral degree	226	17.8
Marital status		
Single	50	3.9
Married	1167	92.0
Divorced	47	3.7
Widowed	4	0.3
Household size		
Up to 3	328	25.9
4	615	48.5
5 and more	325	25.6
Number of children in household		
1	360	28.4
2	663	52.3
3 and more	245	19.3
Healthcare professional		
Yes	165	13.0
No	1103	87.0
Household member is a healthcare professional		
Yes	95	7.5
No	1173	92.5
Total	1268	100.0

**Table 2:** Characteristics of the youngest child referred to in the parental/guardian questionnaire on medication use in an online convenience sample of parents in Serbia.

Characteristic	N	%
Sex		
Male	636	50.2%
Female	632	49.8%
Age category		
Infant (<1 year)	113	8.9%
Toddler (1 to <3 years)	430	33.9%
Preschool child (3 to <6 years)	553	43.6%
Child (6 to <12 years)	172	13.6%
Kindergarten or school attendance		
Yes	1023	80.7%
No	245	19.3%
Chronic illness in the child		
Yes	41	3.2%
No	1227	96.8%
Total	1268	100.0%

**Table 3:** Medication use among children younger than 12 years in an online convenience sample of parents in Serbia.

Medication Use Patterns	N	%
Has your youngest child taken any medication in the past 6 months?		
Yes	900	71.0%
No	368	29.0%
Which of these groups of medications has your youngest child taken in the past 6 months? *		
fever-reducing medication	807	63.6% *
nose drops	534	42.1% *
cough syrup	531	41.9% *
immune system supplements	430	33.9% *
inhalations	597	47.1% *
vitamins and minerals	390	30.8% *
vaccines	191	15.1% *
Has your youngest child taken antibiotics in the past 6 months?		
Yes	693	54.7%
No	575	45.3%

* Multiple-response item. Percentages calculated based on total sample (n = 1268).

**Table 4:** Factors associated with antibiotic use among children younger than 12 years during the previous six months in an online convenience sample of parents in Serbia: multivariable modified Poisson regression analysis (n = 1268).

Characteristic	Adjusted Prevalence Ratio (95% CI)	*p*-Value
Child age		<0.001
Infant (<1 year)	0.48 (0.34–0.68)	<0.001
Toddler (1 to <3 years)	Reference	—
Preschool child (3 to <6 years)	0.85 (0.76–0.95)	0.004
Child (6 to <12 years)	0.63 (0.52–0.77)	<0.001
Child sex		
Male	Reference	—
Female	1.06 (0.96–1.17)	0.228
Parental education		0.682
Primary or secondary education	Reference	—
University education	1.05 (0.94–1.18)	0.395
Postgraduate education	1.02 (0.88–1.19)	0.781
Parent/guardian is a healthcare professional		
No	Reference	—
Yes	0.93 (0.80–1.09)	0.354
Kindergarten/school attendance		
No	Reference	—
Yes	1.20 (1.01–1.43)	0.041
Chronic illness requiring regular medication		
No	Reference	—
Yes	1.51 (1.31–1.75)	<0.001
Number of children in the family		0.036
One	Reference	—
Two	0.88 (0.78–0.98)	0.018
Three or more	0.86 (0.75–1.00)	0.051
Parental age	0.997 (0.99–1.01)	0.607

Adjusted prevalence ratios (aPRs) and 95% confidence intervals (CIs) estimated using modified Poisson regression with robust sandwich standard errors.

**Table 5:** Factors associated with parent-directed medication without physician recommendation among children who received medication during the previous six months in an online convenience sample of parents in Serbia: multivariable modified Poisson regression analysis (n = 900).

Characteristic	Adjusted Prevalence Ratio (aPR)	95% CI	*p*-Value
Child age group			
Toddler	1.00 (reference)	—	—
Infant	0.65	0.44–0.97	**0.036** *
Preschool	1.18	1.04–1.35	**0.014**
School-age	1.04	0.84–1.28	0.731
Child sex			
Male	1.00 (reference)	—	—
Female	1.03	0.92–1.14	0.661
Parental education			
Primary or secondary education	1.00 (reference)	—	—
University education	1.23	1.06–1.42	**0.007**
Postgraduate education	1.27	1.07–1.52	**0.008**
Parent is a healthcare professional			
No	1.00 (reference)	—	—
Yes	1.28	1.12–1.46	**<0.001**
Kindergarten/school attendance			
No	1.00 (reference)	—	—
Yes	1.07	0.87–1.32	0.540
Child has a chronic illness			
No	1.00 (reference)	—	—
Yes	0.60	0.40–0.91	**0.016**
Household size			
≤3 members	1.00 (reference)	—	—
4 members	1.08	0.95–1.23	0.229
≥5 members	0.94	0.79–1.12	0.461
Parental age, per additional year	1.00	0.98–1.01	0.456

* *p* < 0.05.

## Data Availability

The original contributions presented in this study are included in the article/Supplementary Materials. Further inquiries can be directed to the corresponding author.

## Supplementary Materials

The following supporting information can be downloaded at https://www.mdpi.com/article/10.3390/medsci14060624/s1, Table S1: Medication use during the previous six months according to physician recommendation or parent-directed medication without physician recommendation; Table S2: Univariable logistic regression analysis of factors associated with reported antibiotic use in children; Table S3: Characteristics associated with parent-directed medication without physician recommendation of children in univariable logistic regression analysis; Table S4: Sensitivity analyses of factors associated with antibiotic use among children during the previous six months using modified Poisson regression (n = 1268); Table S5: Specifications and model-fit statistics for the principal and sensitivity analyses of antibiotic use among children for modified Poisson regression (n = 1268); Table S6: Diagnostic measures for modified Poisson regression models of antibiotic use; Table S7: Sensitivity analysis of factors associated with antibiotic use among children using multivariable logistic regression (n = 1268); Table S8: Sensitivity analyses of factors associated with parent-directed medication without physician recommendation among children who received medication during the previous six months using modified Poisson regression (n = 900); Table S9: Specifications and model-fit statistics for the principal and sensitivity analyses of parent-directed medication without physician recommendation using modified Poisson regression (n = 900); Table S10: Diagnostic measures for modified Poisson regression models of parent-directed medication without physician recommendation; Table S11: Logistic regression sensitivity analysis of factors associated with parent-directed medication without physician recommendation among medication users (n = 900); Figure S1: Parental interest in receiving education on the safe use of medicines in children reported in an online convenience sample of parents in Serbia.
