# Supplementary material for: Medication and Antibiotic Use Among Children Reported by an Online Convenience Sample of Parents in Serbia: A Cross-Sectional Survey
Source: Med Sci (Basel). 2026 Sep 30;14(6):624. doi: 10.3390/medsci14060624 (PMC13706702; doi:10.3390/medsci14060624)
Supplement: Supplementary file 1 [file medsci-14-00624-s001.zip › medsci-4588561-supplementary.pdf]

Article

Medication and Antibiotic Use among Children Reported by an Online Convenience Sample of Parents in Serbia: A Cross-Sectional Survey

Ana Tomas Petrović [1], Nela Adžić [2], Nebojša Pavlović [3,*], Branislava Teofilović [3], Milica Paut Kusturica [1], Tihomir Dugandžija [4,5], Milica Vasilić [6] and Nebojša Stilinović [1]

[1] Department of Pharmacology, Toxicology and Clinical Pharmacology, Faculty of Medicine, University of Novi Sad, Hajduk Veljkova 3, 21000 Novi Sad, Serbia; ana.tomas@mf.uns.ac.rs (A.T.P.), milica.paut-kusturica@mf.uns.ac.rs (M.P.K.), nebojsa.stilinovic@mf.uns.ac.rs (N.S.)

[2] Faculty of Medicine, University of Novi Sad, Hajduk Veljkova 3, 21000 Novi Sad, Serbia 015855@mf.uns.ac.rs

[3] Department of Pharmacy, Faculty of Medicine, University of Novi Sad, Hajduk Veljkova 3, 21000 Novi Sad, Serbia; branislava.teofilovic@mf.uns.ac.rs

[4] Department of Epidemiology, Faculty of Medicine, University of Novi Sad, Hajduk Veljkova 3, 21000 Novi Sad, Serbia; tihomir.dugandzija@mf.uns.ac.rs

[5] Oncology Institute of Vojvodina, Put Doktora Goldmana 4, 21204 Sremska Kamenica, Serbia [6] Pulmonary Institute of Vojvodina, Put Doktora Goldmana 4, 21204 Sremska Kamenica, Serbia; vasilicmi-ca96@live.com

* Correspondence: nebojsa.pavlovic@mf.uns.ac.rs

Supplementary Materials

Supplementary Materials: The following supporting information can be downloaded at https://www.mdpi.com/article/10.3390/medsci14060624/s1, Table S1: Medication use during the previous six months according to physician recommendation or parent-directed medication without physician recommendation; Table S2: Univariable logistic regression analysis of factors associated with reported antibiotic use in children; Table S3: Characteristics associated with parent-directed medication without physician recommendation of children in univariable logistic regression analysis; Table S4. Sensitivity analyses of factors associated with antibiotic use among children during the previous six months using modified Poisson regression (n = 1268); Table S5. Specifications and model-fit statistics for the principal and sensitivity analyses of antibiotic use among children for modified Poisson regression (n = 1268); Table S6. Diagnostic measures for modified Poisson regression models of antibiotic use; Table S7. Sensitivity analysis of factors associated with antibiotic use among children using multivariable logistic regression (n = 1268); Table S8. Sensitivity analyses of factors associated with parent-directed medication without physician recommendation among children who received medication during the previous six months using modified Poisson regression (n = 900); Table S9. Specifications and model-fit statistics for the principal and sensitivity analyses of parent-directed medication without physician recommendation using modified Poisson regression (n = 900); Table S10. Diagnostic measures for modified Poisson regression models of parent-directed medication without physician recommendation; Table S11. Logistic regression sensitivity analysis of factors associated with parent-directed medication without physician recommendation among medication users (n = 900); Figure S1. Parental interest in receiving education on the safe use of medicines in children reported in an online convenience sample of parents in Serbia.

Table S1. Medication use during the previous six months according to physician recommendation or parent-directed medication without physician recommendation

Medicines	Physician recommendation		Parent-directed medication without physician recommendation		Total**	
fever-reducing medication*	574	71.1%	310	38.4%	807	100.0%
nasal drops*	419	78.5%	150	28.1%	534	100.0%
cough syrup*	410	77.2%	156	29.4%	531	100.0%
immune system supplements*	213	49.5%	219	50.9%	430	100.0%
Inhalations*	519	86.9%	115	19.3%	597	100.0%
vitamins and minerals*	198	50.8%	241	61.8%	390	100.0%
vaccines*	191	100.0%	0	0.0%	191	100.0%
antibiotics	688	99.3%	5	0.7%	693	100.0%

*multiple-responses items; **percentages calculated based on those who reported use of specific category in the previous section of the survey (displayed here in column total).

Table S2. Univariable logistic regression analysis of factors associated with reported antibiotic use in children

Variable	Category	cOR (95% CI)	Overall p-value
**Kindergarten/collective childcare attendance**	No	1.00 (Ref.)	<0.001
	Yes	2.00 (1.51-2.66)	
**Child sex**	Male	1.00 (Ref.)	
	Female	1.09 (0.87-1.36)	0.457
**Child age**	Infant (<1 year)	1.00 (Ref.)	<0.001
	Toddler (1 to <3 years)	5.24 (3.29-8.36)	
	Preschool child (3 to <6 years)	3.83 (2.43-6.04)	
	Child (6 to <12 years)	2.09 (1.24-3.51)	
**Chronic illness in the child**	No	1.00 (Ref.)	0.001
	Yes	4.19 (1.84-9.52)	
**Number of children in family**	1	1.00 (Ref.)	<0.001
	2	0.61 (0.47-0.79)	
	3 and more	0.60 (0.43-0.83)	
**Household size**	Up to 3	1.00 (Ref.)	<0.001
	4	0.59 (0.45-0.77)	
	5 and more	0.58 (0.42-0.79)	
**Parent sex**	Male	1.00 (Ref.)	0.410
	Female	1.31 (0.69-2.47)	
**Parent age**	Per 1-year increase	0.99 (0.97-1.01)	0.193
**Parental education**	Primary or secondary	1.00 (Ref.)	0.545
	University degree	1.14 (0.89–1.47)	
	Postgraduate education	1.15 (0.83–1.59)	
**Marital status**	Single	1.00 (Ref.)	0.010
	Married	0.44 (0.24-0.83)	
	Divorced	0.92 (0.38-2.21)	
	Widowed	Not estimable	
**Parent/guardian is a healthcare professional**	No	1.00 (Ref.)	0.301
	Yes	0.84 (0.61-1.17)	
**Household member is a healthcare professional**	No	1.00 (Ref.)	0.844
	Yes	0.96 (0.63-1.46)	

Table S3. Characteristics associated with parent-directed medication without physician recommendation of children in univariable logistic regression analysis.

Variable	Category	cOR (95% CI)	Overall p-value
**Kindergarten/collective child-care attendance**	No	1.00 (Ref.)	0.002
	Yes	1.74 (1.22–2.48)	
**Child sex**	Male	1.00 (Ref.)	0.802
	Female	0.97 (0.74–1.26)	
**Child age**	Infant (<1 year)	1.00 (Ref.)	<0.001
	Toddler (1 to <3 years)	2.23 (1.24–3.98)	
	Preschool child (3 to <6 years)	3.33 (1.87–5.94)	
	Child (6 to <12 years)	2.31 (1.19–4.51)	
**Chronic illness in the child**	No	1.00 (Ref.)	0.008
	Yes	0.40 (0.21–0.79)	
**Number of children in family**	1	1.00 (Ref.)	0.341
	2	1.03 (0.76–1.39)	
	3 and more	0.79 (0.53–1.17)	
**Household size**	Up to 3	1.00 (Ref.)	0.030
	4	1.08 (0.79–1.48)	
	5 and more	0.70 (0.48–1.00)	
**Parent sex**	Male	1.00 (Ref.)	0.395
	Female	0.74 (0.37–1.47)	
**Parent age**	Per 1-year increase	1.02 (0.99–1.04)	0.218
**Parental education**	Primary or secondary	1.00 (Ref.)	0.003
	University degree	1.63 (1.20–2.22)	
	Postgraduate education	1.73 (1.17–2.55)	
**Marital status**	Single	1.00 (Ref.)	0.654
	Married	1.04 (0.55–1.99)	
	Divorced	0.91 (0.37–2.25)	
	Widowed	0.26 (0.03–2.70)	
**Parent/guardian is a healthcare professional**	No	1.00 (Ref.)	0.006
	Yes	1.79 (1.18–2.72)	
**Household member is a healthcare professional**	No	1.00 (Ref.)	0.881
	Yes	1.04 (0.63–1.71)	

Table S4. Sensitivity analyses of factors associated with antibiotic use among children during the previous six months using modified Poisson regression (N = 1,268)

Characteristic	Model2	Model3	Model4	Model5
**Child age**				
Infant vs. toddler	0.48 (0.34–0.68);	0.42 (0.30–0.58);	—	0.48 (0.34–0.68);
Preschool vs. toddler	0.84 (0.76–0.94);	0.88 (0.79–0.98);	—	0.85 (0.76–0.95);
School-aged vs. toddler	0.62 (0.51–0.75);	0.65 (0.54–0.79);	—	0.62 (0.51–0.76);
**Child sex**				
Female vs. male	—	1.06 (0.96–1.17);	1.06 (0.96–1.17);	1.06 (0.97–1.17);
**Parental education**				
University vs. primary/secondary	—	1.04 (0.93–1.17);	1.10 (0.98–1.23);	1.04 (0.93–1.17);
Postgraduate vs. primary/secondary	—	1.01 (0.87–1.18);	1.09 (0.93–1.26);	1.01 (0.87–1.17);
**Parent/guardian is a healthcare profes-**				
Yes vs. no	—	0.93 (0.80–1.09);	0.92 (0.78–1.07);	0.93 (0.79–1.08);
**Kindergarten/school attendance**				
Yes vs. no	1.20 (1.00–1.42);	—	1.41 (1.20–1.67);	1.20 (1.01–1.43);
**Chronic illness requiring regular medica-**				
Yes vs. no	1.50 (1.30–1.74);	1.51 (1.31–1.74);	1.49 (1.27–1.74);	1.53 (1.32–1.77);
**Number of children in the family**				
Two vs. one	0.88 (0.79–0.98);	0.86 (0.77–0.96);	0.86 (0.77–0.96);	—
Three or more vs. one	0.86 (0.75–0.99);	0.84 (0.73–0.98);	0.88 (0.76–1.03);	—
**Household size**				
Four vs. three or fewer	—	—	—	0.86 (0.77–0.96);
Five or more vs. three or fewer	—	—	—	0.85 (0.74–0.97);
**Parental age, per year**	—	1.00 (0.99–1.01);	0.99 (0.98–1.00);	1.00 (0.99–1.01);

Values are adjusted prevalence ratios (aPRs) with 95% confidence intervals (CIs)

Table S5. Specifications and model-fit statistics for the principal and sensitivity analyses of antibiotic use among children for modified Poisson regression (N = 1,268)

Model	Specification	Residual deviance	Residual df	Pearson χ^2	AIC
1	Principal model	790.75	1,255	575.28	2202.75
2	Reduced model	792.09	1,260	574.74	2194.09
3	Principal model excluding kindergarten/school attendance	792.65	1,256	575.10	2202.65
4	Principal model excluding child age	813.38	1,258	575.14	2219.38
5	Principal model replacing number of children with household size	790.00	1,255	574.86	2202.00

Table S6. Diagnostic measures for modified Poisson regression models of antibiotic use

Model	N	Fitted prevalence, min–max	Fitted prevalence >1, n (%)	Maximum leverage	Standardized Pearson residuals, min–max	Maximum Cook's distance	Cook's distance >4/N, n (%)
1	1,268	0.23–1.11	5 (0.39)	0.0490	−1.02 to 1.64	0.0037	2 (0.16)
2	1,268	0.25–1.10	4 (0.32)	0.0381	−1.00 to 1.51	0.0044	2 (0.16)
3	1,268	0.23–1.07	3 (0.24)	0.0472	−0.99 to 1.63	0.0037	2 (0.16)
4	1,268	0.32–1.07	4 (0.32)	0.0493	−0.96 to 1.20	0.0037	2 (0.16)
5	1,268	0.22–1.14	5 (0.39)	0.0460	−1.03 to 1.65	0.0038	2 (0.16)

Table S7. Sensitivity analysis of factors associated with antibiotic use among children using multivariable logistic regression (N = 1,268)

Characteristic	Adjusted odds ratio (95% CI)	p-value
**Child age**		<0.001
Infant (<1 year)	0.30 (0.17–0.51)	<0.001
Toddler (1 to <3 years)	Reference	—
Preschool child (3 to <6 years)	0.65 (0.48–0.87)	0.004
School-aged child (6 to <12 years)	0.35 (0.23–0.53)	<0.001
**Child sex**		
Male	Reference	—
Female	1.15 (0.91–1.44)	0.246
**Parental education**		0.668
Primary or secondary education	Reference	—
University education	1.13 (0.86–1.47)	0.388
Postgraduate education	1.04 (0.73–1.48)	0.826
**Parent/guardian is a healthcare professional**		
No	Reference	—
Yes	0.86 (0.61–1.21)	0.378
**Kindergarten/school attendance**		
No	Reference	—
Yes	1.61 (1.07–2.42)	0.021
**Chronic illness requiring regular medication**		
No	Reference	—
Yes	4.26 (1.84–9.85)	0.001
**Number of children in the family**		0.036
One	Reference	—
Two	0.71 (0.54–0.94)	0.015
Three or more	0.69 (0.48–0.98)	0.039
**Parental age, per year**	0.99 (0.97–1.02)	0.589

Included observations 1,268; -2 log likelihood 1646.477; Cox and Snell R^2 0.076; Nagelkerke R^2 0.102; Hosmer–Lemeshow χ^2 7.970, df 8, p-value 0.436; Overall classification accuracy (cut-off 0.50) 61.4%

Table S8. Sensitivity analyses of factors associated with parent-directed medication without physician recommendation among children who received medication during the previous six months using modified Poisson regression (N = 900)

Characteristic	Model2	Model3	Model4	Model5
Child age				
Infant vs. toddler	0.65 (0.44–0.98); 0.037	0.62 (0.43–0.90); 0.011	—	0.65 (0.43–0.97); 0.034
Preschool vs. toddler	1.16 (1.02–1.32); 0.020	1.20 (1.05–1.36); 0.006	—	1.19 (1.04–1.35); 0.011
School-aged vs. toddler	1.01 (0.83–1.24); 0.905	1.05 (0.85–1.30); 0.637	—	1.05 (0.85–1.30); 0.669
Child sex				
Female vs. male	—	1.03 (0.92–1.15); 0.643	1.02 (0.91–1.13);	1.03 (0.92–1.15); 0.631
Parental education				
University vs. primary/secondary	1.21 (1.05–1.40); 0.009	1.22 (1.06–1.42); 0.007	1.23 (1.06–1.43);	1.24 (1.07–1.43); 0.004
Postgraduate vs. primary/secondary	1.25 (1.05–1.48); 0.012	1.27 (1.06–1.51); 0.008	1.27 (1.06–1.51);	1.29 (1.08–1.53); 0.004
Parent/guardian is a healthcare profes-				
Yes vs. no	1.28 (1.12–1.46); <0.001	1.28 (1.12–1.46);	1.25 (1.09–1.43);	1.28 (1.12–1.46);
Kindergarten/school attendance				
Yes vs. no	1.07 (0.87–1.31); 0.552	—	1.33 (1.10–1.60);	1.06 (0.86–1.31); 0.569
Chronic illness requiring regular med-				
Yes vs. no	0.60 (0.40–0.91); 0.016	0.60 (0.40–0.91); 0.015	0.61 (0.40–0.93);	0.60 (0.40–0.90); 0.015
Household size				
Four vs. three or fewer	1.08 (0.95–1.22); 0.258	1.08 (0.95–1.23); 0.265	1.09 (0.95–1.24);	—
Five or more vs. three or fewer	0.93 (0.78–1.10); 0.388	0.93 (0.78–1.11); 0.431	0.93 (0.78–1.10);	—
Number of children in the family				
Two vs. one	—	—	—	1.05 (0.92–1.19); 0.458
Three or more vs. one	—	—	—	0.98 (0.82–1.17); 0.805
Parental age, per year	—	1.00 (0.98–1.01); 0.474	1.00 (0.99–1.01);	1.00 (0.98–1.01); 0.447

Values are adjusted prevalence ratios (aPRs) with 95% confidence intervals (CIs)

Table S9. Specifications and model-fit statistics for the principal and sensitivity analyses of parent-directed medication without physician recommendation using modified Poisson regression (N = 900)

Model	Specification	Residual deviance	Residual df	Pearson χ^2	AIC
1	Principal model	551.35	887	385.42	1605.35
2	Reduced model	551.68	889	385.25	1601.68
3	Principal model excluding kindergarten/school attendance	551.51	888	385.50	1603.51
4	Principal model excluding child age	556.98	890	385.41	1604.98
5	Principal model replacing household size with number of children	552.63	887	385.23	1606.63

Table S10. Diagnostic measures for modified Poisson regression models of parent-directed medication without physician recommendation

**Model**	**N**	**Fitted prevalence, min–max**	**Fitted prevalence >1, n (%)**	**Maximum leverage**	**Standardized Pearson residuals, min–max**	**Maximum Cook's distance**	**Cook's distance >4/N, n (%)**
1	900	0.25–0.93	0 (0.00)	0.0472	−0.95 to 1.48	0.0043	0 (0.00)
2	900	0.26–0.91	0 (0.00)	0.0473	−0.96 to 1.46	0.0050	2 (0.22)
3	900	0.25–0.93	0 (0.00)	0.0469	−0.94 to 1.48	0.0044	0 (0.00)
4	900	0.26–0.85	0 (0.00)	0.0439	−0.92 to 1.46	0.0058	2 (0.22)
5	900	0.26–0.92	0 (0.00)	0.0493	−0.93 to 1.44	0.0042	0 (0.00)

Table S11. Logistic regression sensitivity analysis of factors associated with parent-directed medication without physician recommendation among medication users (N = 900)

Characteristic	Adjusted odds ratio (95% CI)	p-value
**Child age**		0.007
Infant (<1 year)	0.45 (0.23–0.91)	0.027
Toddler (1 to <3 years)	Reference	—
Preschool child (3 to <6 years)	1.53 (1.09–2.13)	0.013
School-aged child (6 to <12 years)	1.10 (0.67–1.82)	0.704
**Child sex**		
Male	Reference	—
Female	1.06 (0.81–1.40)	0.659
**Parental education**		0.007
Primary or secondary education	Reference	—
University education	1.59 (1.15–2.21)	0.005
Postgraduate education	1.78 (1.17–2.73)	0.008
**Parent/guardian is a healthcare professional**		
No	Reference	—
Yes	1.98 (1.28–3.06)	0.002
**Kindergarten/school attendance**		
No	Reference	—
Yes	1.17 (0.73–1.89)	0.519
**Chronic illness requiring regular medication**		
No	Reference	—
Yes	0.36 (0.18–0.72)	0.004
**Household size**		0.132
≤3 members	Reference	—
Four members	1.23 (0.88–1.71)	0.224
Five or more members	0.88 (0.59–1.30)	0.505
**Parent age**	0.99 (0.96–1.02)	0.465

Included observations 900; −2 log likelihood 1173.855; Cox and Snell R^2 0.060; Nagelkerke R^2 0.080; Hosmer–Lemeshow χ^2 6.467, df 8, p-value 0.595; Overall classification accuracy (cut-off 0.50) 61.6%

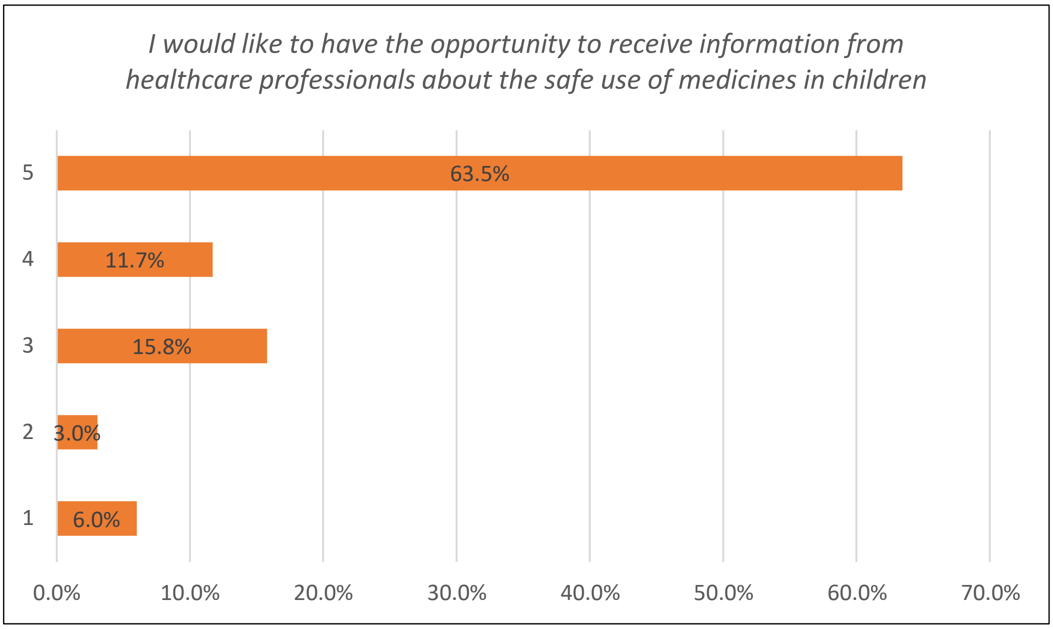

Figure S1. Parental interest in receiving education on the safe use of medicines in children reported in an online convenience sample of parents in Serbia.
